# Physical Activity Enforces Well-being or Shame in Children and Adolescents With Asthma: A Meta-ethnography

**DOI:** 10.1177/00469580241290086

**Published:** 2024-11-05

**Authors:** Thomas Westergren, Hanne Aagaard, Elisabeth O.C. Hall, Mette Spliid Ludvigsen, Liv Fegran, Nastasja Robstad, Åsa Audulv

**Affiliations:** 1 Department of Public Health,University of Stavanger, Norway; 2 Department of Health and Nursing Science,University of Agder, Kristiansand, Norway; 3 Lovisenberg Diaconal University College, Oslo, Norway; 4 Faculty of Health,Aarhus University, Aarhus, Denmark; 5 Faculty of Health Sciences and Nursing,University of Faroe Islands, Torshavn, Denmark; 6 Department of Clinical Medicine-Randers Regional Hospital, Aarhus University, Aarhus, Denmark; 7 Faculty of Nursing and Health Sciences,Nord University, Bodø, Norway; 8 Department of Paediatrics,Sørlandet Hospital HF, Kristiansand, Norway; 9 Department of Nursing,Umeå University, Sweden

**Keywords:** asthma, participation in physical activity, resilience, shame, psychological well-being, meta-synthesis

## Abstract

Asthma symptoms and experiences of dyspnea challenge participation in physical activity (PA). Therefore, in-depth understanding of experiences with PA is essential. In this meta-ethnography, we synthesized published qualitative studies of experiences of children and adolescents with asthma that influenced, or limited, participation in PA. We followed Noblit and Hare’s 7 phases of meta-ethnography. We searched relevant databases by December 18, 2023 for published peer-reviewed studies (Medline (OVID), Embase (OVID), PsycINFO (OVID), CINAHL (EBSCHOhost), SPORTDiscus (EBSCHOhost), SocINDEX (EBSCHOhost), and Social Science Citation Index (WoS)) and theses (ProQuest Nursing & Allied Health Source, ProQuest Healthcare Administration Database, and ProQuest Public Health Database). We conducted study selection and assessment of methodological quality and data extraction using Joanna Briggs Institute’s methodology. Sixteen reciprocally related qualitative studies, representing experiences of 238 children and adolescents aged 4 to 18 years were included. We translated primary study concepts and findings into 3 themes covering relationships with others, emotions, and behaviors related to PA participation: (1) feeling related to and connected with friends and family in PA; (2) acquiring and managing new PA and asthma skills; and (3) enjoying PA and experiencing well-being. We also defined 3 themes covering aspects related to PA limitations: (4) feeling misunderstood and penalized in relation to PA; (5) experiencing nervousness, embarrassment, shame, and sadness during PA; and (6) withdrawing from PA due to asthma, environment, and/or socially imposed attitudes. The themes were synthesized into the following lines of argument: children and adolescents with asthma experience that PA enforces empathic/non-empathic relationships, vulnerability, and awareness; PA enhances resilient participation and well-being, or reinforces resignment to isolation and shame. From the outset of either relatedness or being penalized, youngsters with asthma either manage well and experience well-being, or experience shame and withdrawal.

**Registration:** PROSPERO No. 164797

**What do we alread know about this topic?**

Asthma may challenge participation in physical activity, whereas physical activity constitutes a possibility for improved health, community, and belonging in children and adolescents with asthma.

**How does the meta-ethnography contribute to the field?**

The meta-ethnography contributes with novel insights about how physical activity, as experienced by children and adolescents withs asthma, may enhance resilient participation, relatedness, and well-being as well as reinforce resignment to withdrawal, isolation, and shame. Pathways were induced from either emphaty and relatedness, or feeling misunderstood and penalized.

**What are the implications towards theory, practice, and policy?**

Increased asthma knowledge and awareness are needed among sport coaches, teachers, parents, and children and adolescents in general. Initiatives to meet this need could benefit from incorporating how shame and resilience develop in humans, and in collaboration with schools, school health services, families, children, adolescents, and organized sports entities.

## Introduction

In childhood, asthma is the most common chronic lower respiratory disease.^1^ “Asthma is a chronic inflammatory disorder, characterized by varying airflow obstruction, bronchial hyperresponsiveness, and recurrent episodes of wheeze, cough, shortness of breath, and chest tightness”^1^ (p. 978). Due to its symptom presentations, asthma may interfere with PA,^2^ defined as any bodily movement produced by the contraction of skeletal muscles that increases energy expenditure above the resting level.^3^ About 20%^2^ to 50%^4^ of children with asthma may perceive limitations of physical activity (PA). PA may include exercise, physical education, organized and non-organized sports, play, transportation, or physical labor. Increased energy expenditure through PA may, in all humans, create respiratory discomfort, limb muscle discomfort, and general exertion.^5^ Both general and asthma benefits of PA and increased fitness, are established as state-of-the art.^6^ Less controlled asthma, referring to manifestations of the disease not well managed by treatment,^7^ more severe bronchial hyperresponsiveness, allergy, and overweight seem to be associated with an increased perceived limitation of PA.^2^ By contrast, increased asthma control is reported in association with increased PA and physical fitness.^8^ Consequently, PA is both a challenge and a possibility for improved health in children suffering from asthma. The literature about management of asthma and PA in children points at parent and child misinterpretations and overprotection as reasons for not using the benefits of PA.^9^ PA perceptions of children with asthma, however, develop continuously through experiences and interactions with their surroundings.^10^

Dyspnea, defined as “a subjective experience of breathing discomfort that consists of qualitatively distinct sensations that vary in intensity,”^11^ is similarly described by children with asthma as by adults, and the experience varies with disease severity.^12^ The dyspnea experience may, however, be perceived as a normal reaction to physical exertion as well as to symptoms related to a disease.^13^ In a qualitative study of school children with asthma.^14^ children overwhelmed by dyspnea shared that they were restricted in activities, illustrated by a quote of “not being a player.”

Recently, a scoping review on aspects beyond physiological restrictions summarized subjective experiences in relation to PA participation among children and adolescents with asthma.^15^ Intrapersonal, interpersonal, and environmental factors were reported to correlate with levels of PA or were experienced in relation to participation.^15^ Social discomfort, exceeding the physical discomfort of breathlessness, was reported,^16^ as was enjoyment, physical self-concept, social support,^17^ attitudes and beliefs,^18^ and home environment.^19^ Participation in PA also represented a sense of community and belonging.^20^

From qualitative findings, we know that seasonal variations may present experienced challenges for PA,^21^ and from cross-sectional quantitative studies we know that asthma symptoms and exacerbations vary with season and geographic locations.^22^ Moreover, both the disease itself and disease management vary with age, gender, and developmental stages.^23-25^ Current understanding of participation in, and limitation of PA, in children and adolescents with asthma is hence related to variable disease symptoms, growth and development, as well as several biopsychosocial aspects and shifting environments.

### The Rationale for the Review: Phase 1 of the Meta-Ethnography

To better describe PA experiences and perceived limitations during childhood and adolescence, an in-depth interpretive synthesis of qualitative findings is needed. We conducted preliminary searches to rule out existing reviews, registered with PROSPERO (No. 164797), and published a protocol.^26^ Although the relation between asthma and PA has been well investigated, and several qualitative studies on the topic exist, we found no systematic review exploring and synthesizing the subjective experiences of children and adolescents with asthma concerning PA. A novel conceptual understanding of the subjective perspectives of children and adolescents themselves may contribute to better efforts to include them in school, leisure activities, and organized PA. Therefore, the objective of this meta-ethnography was to synthesize published qualitative studies about experiences of children and adolescents with asthma related to participation in, or limitations of, PA.

## Materials and Methods

We conducted a meta-ethnography following 7 phases as described by Noblit and Hare.^27^ Initially, we planned a meta-aggregation synthesis according to Joanna Briggs Institute (JBI) methods.^28^ However, we chose meta-ethnography to obtain a more interpretative exploration across the existing literature.^27^

### Authors’ Background and Presuppositions

All authors have a professional nursing background, are experienced qualitative researchers, and are familiar with various meta-synthesis methods and with literature concerning children and adolescents with asthma. To maintain reflexivity, all authors discussed each phase, and scrutinized choices, translations, interpretations, synthesis, reporting, and illustrations.

### Deciding What Was Relevant: Phase 2 of the Meta-Ethnography

#### Inclusion criteria

We considered studies of (1) children and adolescents 6 to 18 years of age, with a clinical and/or self-/parent-referred diagnosis of asthma as research participants; (2) concerning participants’ subjective experiences related to participation in, or limitation of, PA; (3) in studies originating from all contexts and countries; (4) with descriptive, explorative, and evaluation designs that focused on qualitative data including, but not limited to, designs such as phenomenology, grounded theory, ethnography, action, or feminist research. Studies indexed in relevant databases and published with the full text in English were considered, unrestricted by year of publication.

Studies including parent- or clinician-reported experiences in addition to those of children with asthma were included if experiences of children and/or adolescents were separable for data extraction. We considered only studies that in the aim explicitly addressed PA experiences such as exercise, sports, and other possible forms of PA.

#### Search strategy

We undertook initial limited searches in MEDLINE and CINAHL followed by analysis of the words contained in titles and abstracts, and in index terms used. This strategy further informed our development of a final search strategy tailored for each information source. The full database search is detailed in Supplemental File 1, which we produced in collaboration with a research librarian. In all included studies, we conducted backtracking of references and forward citations searches in ISI Web of Science, Scopus, and Google Scholar. Documentation relating to reference and citations searches is detailed in Supplemental File 2.

The following databases were searched by December 18, 2023:

Medline (OVID), Embase (OVID), PsycINFO (OVID), CINAHL (EBSCHOhost), SPORTDiscus (EBSCHOhost), SocINDEX (EBSCHOhost), and Social Science Citation Index (WoS).

The search for unpublished studies to identify theses and dissertations was carried out by December 18, 2023, in ProQuest Nursing & Allied Health Source, ProQuest Healthcare Administration Database, and ProQuest Public Health Database.

#### Study selection

Following the search, we collated all identified records, uploaded into Rayyan QCRI online software,^29^ and removed duplicates. Two reviewers independently screened titles and abstracts (first author and a share to each co-author), and subsequently full texts, for assessment against the inclusion criteria for the review. We excluded full text reports that did not meet the inclusion criteria and provided reasons for exclusion. We resolved disagreements between the reviewers through discussion in the group.

### Reading Included Studies; Phase 3 of the Meta-Ethnography

Included reports were read iteratively for familiarization, assessment of quality, and data extraction. Two reviewers independently and critically appraised selected reports (first author and a share to each co-author) for methodological quality using the JBI Qualitative Assessment and Review Instrument.^28^ We resolved disagreements between the reviewers through discussion within the group. Two reviewers independently (first author and a share to each co-author) extracted qualitative data from reports using the standardized JBI data extraction tool.^28^ Extracted data included details about the population (age, gender, asthma diagnosis criteria, disease severity, symptoms presentation), context (season, PA/recruitment/social setting, culture, geographical location), study methods, and the phenomena of interest relevant to the review questions.^28^ We further extracted and coded findings with concepts and wording close to participants’ own words using NVivo Pro software, Release 1.7 (QSR International, Burlington, Massachusetts).

### Determining How Studies Were Related: Phase 4 of the Meta-Ethnography

Phase 4 of the meta-ethnography includes listing, juxtaposing, and grouping studies to identify commonalities, differences, and relationships between studies and their findings.^27^ To determine the relationship between studies, we grid tabulated concepts based on coding in NVivo describing findings from each included study. As suggested by France et al^30^ we then grouped concepts. First, we grouped concepts related to our aim, that is, those concerning experiences with participation in PA, and those concerning limitations of PA. Within those categories of concepts, we identified and grouped concepts describing accounts concerning: emotions reported, relationships, and management of asthma and PA reciprocally. Next, we grouped studies into 3 categories based on our interpretations while reading and coding. One group represented findings primarily describing that asthma restrains PA (n = 4), another represented findings that asthma does not restrain PA (n = 5), and a third represented both perspectives (n = 7). At this stage, we made an initial interpretation that studies—although different and refutational in their emphasis on participation versus limitation—were reciprocally related, and they represented accounts of experiences concerning participation and limitation across studies, study contexts, and age ranges. We acknowledged methodological differences between studies. Moreover, we assumed that studies could be well related, including both first- and second-order interpretations of participants’ voices. To avoid reproducing equivocal interpretations, we evaluated whether interpretive studies that did not satisfy item 7 on the critical appraisal (no reporting of the influence of the researcher on the research, and vice versa), were reporting incongruent findings compared with other included studies. Then, we listed studies and sorted them by the account of participants’ age, starting with the study including the youngest participant^31^ as a starting point of translation between studies to explore whether age of participants could account for moving from limitation to participation.

### Translating Studies Into One Another: Phase 5 of the Meta-Ethnography

In Phases 3 and 4, we came aware of which studies reported rich findings concerning participation and limitation, and which studies reported more limited findings concerning the phenomenon of interest. We recognized particularly that the study by Cardwell and Elliott,^21^ concerning nuances of participants’ experiences, was informing all themes and concepts in the grid. Hence, we chose this study as the index study for translation, as suggested by Noblit and Hare.^27^ We treated grid concepts as first- and second-order constructs indifferently, as suggested by France et al^30^ Starting with the index study,^21^ we identified each concept/nuance and compared it with the rest of the included study findings going through each, finding by finding, and each study, starting with the study that included the youngest participants^31^ until we compared the study with the oldest participants.^32^ Each concept, representing first- and second-order interpretations, then informed the evolving translations, which according to Noblit and Hare^27^ and France et al,^30^ should embody more than each study does alone. Our translation, based on the constant comparative method and the account-by-account translation as described above,^27^ resulted in 6 themes representing relationships, emotions, and behaviors, related to participation in PA (three translations/themes) and experienced limitations of PA (three separate translations/themes).

### Synthesizing Translations: Phase 6 of the Meta-Ethnography

In Phase 6, we interpreted the findings across studies, represented by our themes into a whole conceptual understanding.^27^ As our aim was twofold, focusing both on PA participation and limitations of PA, we chose interpretation and synthesis of each perspective before developing a lines-of-argument synthesis representing the storyline.^27^ To acknowledge that participation and limitation may be related in participants’ lived experiences, and that emotions, relationships, and management of disease and activities may interact within daily experiences, we looked for commonalities and associations among translations while synthesizing.

At this analysis point, we recognized a pattern from our inductive and data-driven approach—the shame resilience theory, where Brown^33^ describes the continuum of being trapped, isolated, and powerless in shame—and the opposite: feeling connected, empowered, and free in experiencing empathy. Therefore, we chose to use this theory for guidance in developing our lines-of-argument synthesis. Our conceptualization of the whole, representing participants’ experiences were hence synthesized and interpreted.

The expression of the synthesis, starting with search and eligibility results and study characteristics, is outlined below as the seventh and last phase of this meta-ethnography.^27^ Following France et al,^34^ we reported in accordance with the eMERGe guidelines.

## Results

### Search Results

**Figure 1.**
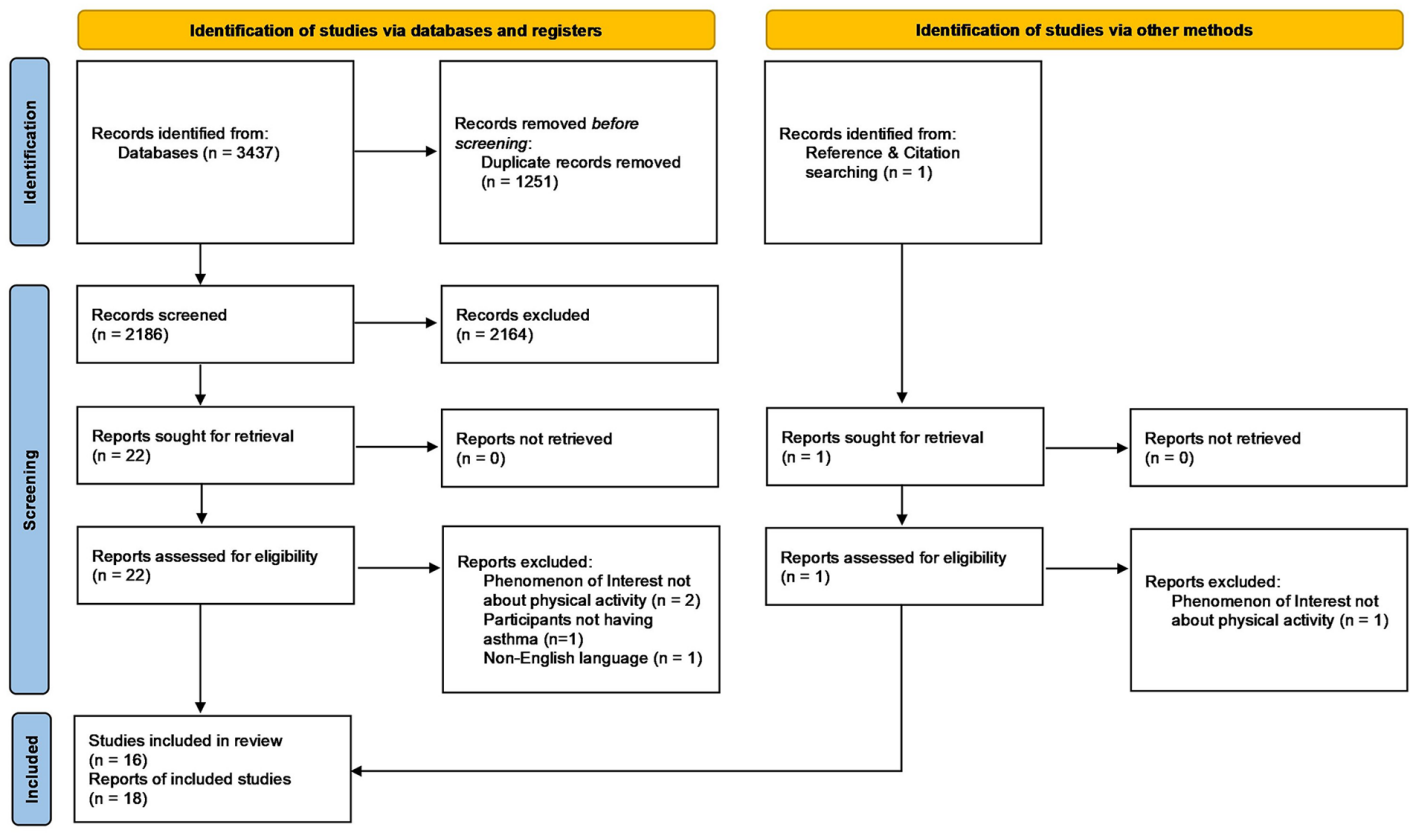
PRISMA flow diagram illustrating the identification, screening, and inclusion results.^40^

The results of the search are reported in full and presented in Figure 1. Twenty-two records from the database search, and 1 identified from citation searching, were evaluated in full text. Two full texts from the database search^35,36^ and the 1 from citation searching^37^ did not address PA in the phenomenon of interest, 1 did not separate children and/or adolescents with asthma from those without,^38^ and 1 was written in Chinese.^39^ Sixteen studies represented by 18 reports were included.

### Characteristics of Included Studies

The included reports/studies were published in the period 2009 to 2023 and originated from Australia,^31^ Canada,^21,41-44^ the US,^10,32,45-48^ UK,^20,49-51^ and Norway.^52,53^ Study and/or recruitment settings ranged from exercise and sports settings,^21,42,47,50,52,53^ schools,^46^ health care services,^20,31,32,41,43,44,49^ home environment,^10,45,48^ and local communities and social media.^32,51^ Data were collected through focus groups^31,41,43,50,52,53^ and individual semi-structured interviews.^10,20,21,32,42,44-49,51^ The studies reported on experiences of a total of 238 children and adolescents with asthma aged 4 to 18 years ranging from mainly a descriptive reporting^21,32,46,47,49-51,53^ to a merely interpretive analysis and reporting.^10,20,31,41-45,48,52^ As stated in the protocol,^26^ we intended to include children from age 6 years as we did not expect younger children to share their experiences on the topic with researchers. One study, however, included some participants from age 4 years,^31^ and we chose to include this study as it did not alter our aim. Most studies were analyzed inductively; however, four studies approached analysis deductively.^21,50,51,53^ Description of the included studies are given in Supplemental File 3, Table 1.

All studies were appraised as being of good quality, although four^46,47,50,51^ did not locate the researcher culturally or theoretically, and eight^20,21,46-51^ did not address influence of the researcher on the research and vice versa, whereas this matter was described in the originating theses of Protudjer^43,45^ but not in the subsequently published papers.^10,41^ Results from critical appraisal of the studies are given in Supplemental File 3, Table 2.

To a large degree, the included studies built on a presumption that asthma may challenge or delimit participation in PA. Concurrently, all studies included either a narrative of participation in PA despite asthma, or of the urge for or strategies to increase participation. Six studies (seven reports),^10,21,32,45,50,52,53^ according to our interpretation, reported a balanced view of possibilities for participation in PA and limitations, whereas 5 studies had a dominating perspective of asthma as a restrictor of PA participation.^20,44,46,49,51^ Five studies (six reports) explicitly reported that asthma does not restrict participation in PA^31,41-43,47,48^ (see Supplemental File 3, Table 3).

### Expressing the Synthesis: Phase 7 of the Meta-Ethnography—Experiences of Participation in, and Limitations of, PA

The translation of included studies and findings into each other, although described with variations in wordings/concepts, methods and level of interpretations, and quality, were reciprocal. The studies described experiences of resilience and participation in PA as well as shame and limitations of PA, illustrated by the following themes: (1) PA enables an emphatic relationship with others, asthma, and self; and (2) PA disables an empathic relationship with others, self, and asthma. In Supplemental File 3, Table 3, we present a grid of primary study authors’ first- and second-order interpretations extracted as codes/concepts from each study related to each of our translations.

### PA Enables an Emphatic Relationship With Others, Asthma, and Self

Considering an interplay between the 3 translations of PA participation, (1) feeling related to and connected with friends and family,^10,20,21,31,32,42,44,45,47,48,52,53^ (2) acquiring and managing new PA and asthma skills,^10,20,21,31,32,41,43-45,47,49-52^ and (3) enjoying PA and experiencing well-being,^10,20,21,31,32,41,43-45,47,50,52,53^ the interplay of emphatic relationships with others, with activities of managing asthma and PA, and with oneself and with one’s emotions, becomes evident as a driver for increased PA participation and an illustration of resilience. Our interpretations were that participants’ experiences were ordered from the outset of social relationships. As developing children, they related to family members, peers, teachers, and coaches in PA, which they also experienced as important in managing asthma and PA. Next, in acquiring and managing skills, they felt supported, and relied on emphatic relationships with people who included and treated them well. Those experiences subsequently nourished possibilities for experiencing joy and well-being, and allowed them to flourish in and through PA.

Through relationships with others in PA, participants experienced protection and support,^31,32,48,53^ as well as awareness and recognition,^20,21,32,53^ and feeling related to and connected with friends and family.^10,20,42,44,45,47^ In sharing their knowing,^10,32,45,48,53^ which is more of a “how to,” and a broader term than “knowledge about something,” for instance about asthma management, sharing humor and laughter,^52^ and by changing/adjusting,^10,20,21,31,32,41,43-47,49-52^ participants acquired skills to normalize their PA behavior.^10,44,45^ Normalizing was also a process of demystifying and contextualizing self, asthma, and PA participation. In other words, participants’ experiences of relatedness in and through PA participation increased their understanding,^10,20,32,41,43,45,53^ and made them free,^32^ empowered,^41,43,53^ and connected in managing both PA and asthma.^44^ Thus, through empathy from and with others as well as with their chronic disease and the PA setting/situation, they opened themselves for resilience, for joy, and for well-being, through participation in PA.^10,20,21,31,32,41,43-45,47,50,52,53^ As stated by one participant:

*If I’m stressed, like school . . . that might aﬀect how I play. . . But usually when I do play and I’m stressed, when I go out and play, it just goes away. . . So it actually kind of helps me get rid of that* ^21^ *(p. 656).*

### PA Disables an Empathic Relationship With Others, Self, and Asthma

Paradoxically, when children and adolescents with asthma experienced social exclusion, stigmatization, and penalization from peers, teachers, and coaches in PA,^20,21,31,50,51^ a process of limiting PA became visible. The negative treatment from others in relation to asthma and PA created inner negative experiences^10,20,21,32,44-49,52,53^ including nervousness, embarrassment, shame, and sadness, and in the end dislike of moving their own body and tackling mental health challenges.^32^ Experiences following penalization and shame were withdrawal from PA due to several reasons directly or indirectly related to asthma.^10,20,21,31,32,44-47,49-53^ In other words, participants’ unfavorable experiences decreased their choices of reaching out to others for support and increased their loneliness, which again limited their possibilities for favorable change. Reasons for withdrawing include symptoms or fear of symptoms, limitations of access to medication, fear of side effects of medications, hospitalization, uncomfortable environments, or triggers thereof, as well as experiences of physical exertion directly related to the respiratory system or other bodily sensations. Obstacles for PA shared with healthy children were also experienced, including screen time,^41,43^ family issues, online schooling, unsafe neighborhoods, lack of time, and poor sleep.^32^ Participants shared experiences of avoiding PA due to the unease of exertion or breathlessness either by lack of knowledge,^20^ individual beliefs of tolerance,^10,45^ or by conscious choice using asthma as an excuse.^50^ Experiences of explicit emotional reasons were also shared: participants withdrew from PA due to feelings of, and to avoid, being different,^10,45^ due to losing lead in PA competitions, and due to decreased enjoyment.^52^ Consequently, children and adolescents with asthma had several reasons to avoid and withdraw from PA, both related to asthma and in general. Hence, a reinforcing process of individualizing (trying to manage alone with secrecy and silence) and pathologizing (asthma limits PA) fed their shame.

The results of our interpretative translations concerning the disabling experiences and processes of participants in relation to limitations of PA, as outlined above were ordered as follows: (1) feeling misunderstood and penalized in relation to PA; (2) experiencing nervousness, embarrassment, shame, and sadness during PA; and (3) withdrawing from PA due to asthma, environment, and/or socially imposed attitudes. Non-empathic relationships with others, self, and asthma interplayed toward reduced, or limited, PA. Below, a few descriptions from studies are given:

*Participants also explained that low mood, including sadness, depression, and feeling down, negatively impacted adolescent PA engagement* ^32^ *(p. 713)*

*If I am alone and the others are much fitter than me, exercising is not fun* ^53^ *(p. 1251)*

*. . . restricted their activity to avoid being different or “slow.”* ^10^ *(p. 236)*

### The Lines-of-Argument: PA Enforcements of Resilience and/or Shame

**Figure 2.**
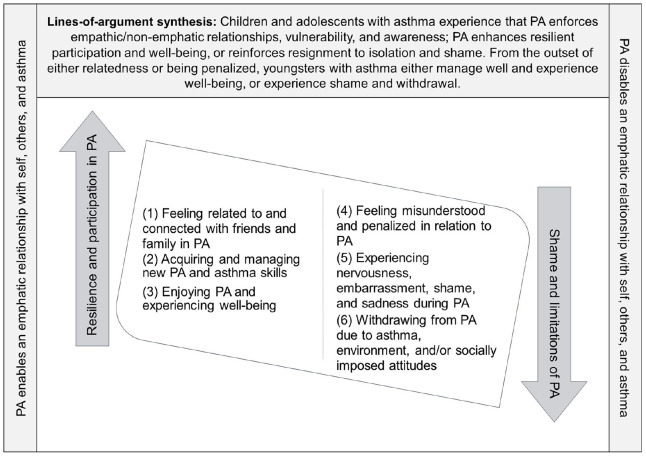
Translations/themes, and the lines-of-argument synthesis across the included studies.

In searching for a storyline and novel conceptual understanding representing the core of participants’ experiences of participation in, and limitations of, PA across the 16 included studies, we formulated the lines-of-argument synthesis. The 6 translations/themes across the included studies, the themes representing participation/empathy/resilience and limitation/shame, as well as the lines-of-argument synthesis are presented in Figure 2.

## Discussion

Within the last 15 years, 238 participants between the ages of 4 and 18 years with asthma have shared their experiences with having the disease and participation in PA, sports, active play, or exercise, and limitations thereof. Drawing on the shame resilience theory by Brown,^33^ we suggest that participation in PA for children and adolescents with asthma put acknowledged vulnerability, critical awareness, and mutual emphatic relationships at stake. PA creates opportunities for wholehearted and active living while having asthma, but also creates the possibility for withdrawing in shame and judging themselves for not being good enough in either the eyes of others or themselves. PA therefore seems to be an activity for children and adolescents with asthma that sharpens the edge on which they must balance in managing asthma, self, and everyday activities. PA hence constitutes an opportunity for healthy development beyond physical fitness and reduced asthma symptoms, as well as the pitfall of being trapped, powerless, and isolated in shame. With use of concepts from Brown,^33^ their relationships with others, self, and PA could be shaped by recognition, awareness, protection, and support, or alternatively with confusion, judgment, fear, anger, and blame.

Brown^33^ illustrates how shame appears in relation to a web of social expectations about what, who, and how one should be, which may leave the individual trapped, powerless, and isolated if one cannot manage the expectations well. While such expectations were not explicitly explained by participants in our included studies, they were implicit. In studies having a dominating perspective that asthma does not restrain PA, ideals that “asthma isn’t an excuse” ^41^ (p. 496), or “there is nothing I can’t do”^31^ (p. 1), illustrate such implicit ideals. On the other hand, in studies having a dominating perspective that asthma restrains PA, such ideals were out of reach as illustrated by statements such as “has asthma . . . so she can’t play”^46^ (p. 811) and ‘I tell them it is my asthma they [my friends] go ‘Oh you’re rubbish”^20^ (p. 324). Studies also contributed with examples of “ideals” that PA was not safe,^20^ making the overall expectations web contradictory concerning PA participation. Participants were expected to do and participate in PA, either by managing to “normalize”^10,44,45^ or by not adjusting well and being penalized.^20,21,31^ Following Brown’s^33^ suggestion about “speaking shame,” acknowledging one’s vulnerability, being critically aware, and reaching out to others, it may seem that children and adolescents with asthma who have acquired such intentions and behavior are those who also manage, enjoy, and benefit from PA. Ciccone,^54^ however, warns about uncritically applying this theory of “vulnerable resilience.” Psychologically, although it may be a powerful tool for increasing resilience, it has strong neoliberal connotations placing the responsibility for self-improvement on the individual rather than on the dominating structures. Consequently, the responsibility of the marginalized (in the current study, children and adolescents with asthma who are limited from PA participation) is to manage “bounce back” themselves.^54^ Our findings point in a different direction than the individual’s responsibility alone. Either participation in PA or limitation of PA seems to start with the social structure in which children and adolescents with asthma live. By feeling related to and connected with, preconditions for empathy with oneself and from others, as well as “speaking shame” and its related concepts/processes are established. Alternatively, by misunderstandings and penalization, the preconditions of shame and its related aspects of confusion, judgment, fear, anger, blame, pathologizing, individualizing, negative reinforcement, isolation, limited understanding and options, and feeling trapped and powerless^33^ may set the agenda. Previous research also aligns with this interpretation; intra- and interpersonal factors and aspects, as well as the environment correlate with level of PA in this population.^15^ We therefore argue that the shame–resilience theory^33^ could be fruitful to understand and enhance PA participation in children and adolescents with asthma, but not primarily by placing the responsibility on the individual with asthma. The theory is useful because it focuses on and guides facilitation of PA contexts to be inclusive, emphatic, aware, and demystified for children and adolescents with, as well as without, asthma. Following the voices and experiences of participants in the included studies, teachers, coaches, and peers may suffer from a lack of asthma awareness.^20,21,31,50,53^ Enhanced education about, and awareness of, asthma in organized sports and schools as well as common understanding of “vulnerable resilience”^33,54^ may create structural preconditions for increased PA participation and enhanced well-being in children and adolescents with asthma.

Our findings and interpretations may be further enlightened by the relationship between self-esteem and shame. Although not specifically about participants with asthma, Budiarto and Helmi^55^ analyzed this relationship across 18 studies, and found that shame was strongly and negatively correlated with self-esteem, and grew stronger with increased age. Their meta-analysis relied on definitions of self-esteem placing it as a human need, a person’s belief in one’s worthiness to be rejoicing and ability to cope, and which is determined by self-assessment (positive and negative) in comparison with others.^55^ Both the process enhancing participation in PA as well as the process limiting participation correspond well to these aspects of self-esteem including the need, the beliefs, and the self-assessment which are all at stake for the participants in our included studies. Previous research shows that both asthma and asthma management vary with age, gender, and developmental stage.^23-25^ and we may expect that management of social relationships and PA participation do so as well. Towns and van Asperen^56^ describe how chronic diseases may create a distortion of body image and isolation from peers impacting development negatively early in adolescence, while increased dependency, less peer acceptance, reduced vocational options, and concerns about relationships follow later. Our current findings alter such a determined pathway, as the participants across studies and ages were well represented in both the participation and resilience paths, as well as the shame and isolation path concerning PA participation. Nevertheless, taking note of how shame and low self-esteem relate more strongly with increased age, the window of opportunity to enhance resilience might be greater in younger ages. Hence, a culture of overprotection,^20^ lack of access to medications,^46^ and lack of asthma awareness^20,21,31,50,53^ may threaten the initiation of opportunities for learning and development of resilient participation. As Woodgate^14^ reported, the experience of dyspnea, which is common in asthma, may lead to feeling different and “not being a player.” In children and adolescents, in particular the younger ones, the responsibility of managing a chronic disease should be shared with parents and the social context of the youngsters.^57^ Hence, exercising responsibility for asthma and PA with empathy and awareness should not be a responsibility for children and adolescents with asthma alone, as peers, parents, teachers, coaches, and healthcare professionals may influence the pathways to either shame and isolation, or participation, resilience, and well-being.

### Strengths and Limitations

The current meta-ethnography was strengthened by a comprehensive and systematic search, and a blinded screening for eligibility, data extraction, and critical appraisal by 2 reviewers independently. The thorough analysis and synthesis, discussed within our well-experienced research group for each phase, also strengthened the synthesis. We have relied on the acknowledged methodology of Noblit and Hare.^27^ The eMERGe reporting guidelines for meta-ethnographies^34^ helped ensure transparency. The findings align with previous knowledge, while adding novel interpretations and conceptualizations. Our synthesis is limited to primary studies published in the English language. Although asthma is a global disease,^1^ and we may expect PA experiences to have commonalities across the globe, our interpretations entirely rely on studies conducted in Western countries, which may not be transferable uncritically to contexts and populations beyond. We also acknowledge that interpretative analysis and synthesis might have been colored by our pre-understanding, although we have made efforts to transparently report our positioning throughout the study as well as our reflections and choices.

## Conclusion

Children and adolescents with asthma experienced pathways of increased PA participation starting with feeling connected and able to relate to friends and family in PA, in which they acquired and managed new asthma and PA skills, subsequently enjoying PA and experiencing well-being. In contrast, the experienced pathways of PA limitations started with being misunderstood and penalized in relation to PA followed by experiencing nervousness, embarrassment, shame, and sadness. Subsequently, they withdrew from PA due to asthma, environment, and/or socially imposed attitudes. Our lines-of-argument synthesis across the included studies contributes with the following novel and conceptual understanding: children and adolescents with asthma experience that PA enforces empathic/non-empathic relationships, vulnerability, and awareness; PA enhances resilient participation and well-being, or reinforces resignment to isolation and shame. From the outset of either relatedness or being penalized, youngsters with asthma either manage well and experience well-being, or experience shame and withdrawal.

Implications for practice will be to develop increased asthma knowledge and awareness among organized sports coaches, teachers (particularly physical education teachers), parents, and children and adolescents in general. Further research should focus on how shared responsibility, asthma knowledge, and awareness can be increased in collaboration with school health services, families, children, and adolescents, and organized sports entities. We suggest informing such efforts also by incorporation of how shame and resilience develop in humans.^33^

## Supplemental Material

sj-docx-1-inq-10.1177_00469580241290086 – Supplemental material for Physical Activity Enforces Well-being or Shame in Children and Adolescents With Asthma: A Meta-ethnography Supplemental material, sj-docx-1-inq-10.1177_00469580241290086 for Physical Activity Enforces Well-being or Shame in Children and Adolescents With Asthma: A Meta-ethnography by Thomas Westergren, Hanne Aagaard, Elisabeth O.C. Hall, Mette Spliid Ludvigsen, Liv Fegran, Nastasja Robstad and Åsa Audulv in INQUIRY: The Journal of Health Care Organization, Provision, and Financing

sj-docx-2-inq-10.1177_00469580241290086 – Supplemental material for Physical Activity Enforces Well-being or Shame in Children and Adolescents With Asthma: A Meta-ethnography Supplemental material, sj-docx-2-inq-10.1177_00469580241290086 for Physical Activity Enforces Well-being or Shame in Children and Adolescents With Asthma: A Meta-ethnography by Thomas Westergren, Hanne Aagaard, Elisabeth O.C. Hall, Mette Spliid Ludvigsen, Liv Fegran, Nastasja Robstad and Åsa Audulv in INQUIRY: The Journal of Health Care Organization, Provision, and Financing

sj-docx-3-inq-10.1177_00469580241290086 – Supplemental material for Physical Activity Enforces Well-being or Shame in Children and Adolescents With Asthma: A Meta-ethnography Supplemental material, sj-docx-3-inq-10.1177_00469580241290086 for Physical Activity Enforces Well-being or Shame in Children and Adolescents With Asthma: A Meta-ethnography by Thomas Westergren, Hanne Aagaard, Elisabeth O.C. Hall, Mette Spliid Ludvigsen, Liv Fegran, Nastasja Robstad and Åsa Audulv in INQUIRY: The Journal of Health Care Organization, Provision, and Financing
