# Supplementary material for: Physical Activity Enforces Well-being or Shame in Children and Adolescents With Asthma: A Meta-ethnography
Source: Inquiry. 2024 Nov 5;61:00469580241290086. doi: 10.1177/00469580241290086 (PMC11536505; doi:10.1177/00469580241290086)
Supplement: sj-docx-1-inq-10.1177_00469580241290086 – Supplemental material for Physical Activity Enforces Well-being or Shame in Children and Adolescents With Asthma: A Meta-ethnography [file sj-docx-1-inq-10.1177_00469580241290086.docx]

**EMBASE (Ovid): Embase <1974 to 2023 Week 50> Date: 18.12.2023**

Link, access is needed: <https://ovidsp.ovid.com/ovidweb.cgi?T=JS&NEWS=N&PAGE=main&SHAREDSEARCHID=5N4qWaTFCsoXZVjNMHC8ajEq1O869hRAAUpKGRwAdezzm0qhMl7XiCH80phAYZ20h>

1 boy/ or child/ or girl/ or juvenile/ or preschool child/ or school child/ or toddler/ or adolescent/ or adolescence/ 3349929

2 (adolescen* or boy or boys or child* or girl* or girls* or juvenil* or kid or kids or schoolchild* or teens or teenager* or young* or youth*).ti,ab,kf. 3462506

3 1 or 2 4807231

4 asthma*.ti,ab,hw,kf. 346271

5 exp physical activity/ or motor activity/ 589447

6 exp exercise/ 439881

7 exp sport/ 215845

8 (sedentar* or "screen time").ti,ab,hw,kf. 66594

9 inactiv*.ti,ab,kf. 409138

10 (exercise* or "motor activ*" or "physical activ*" or play or playing or playtime or sport* or training).ti,ab,kf. 2463781

11 or/5-10 3287790

12 qualitative research/ or hermeneutics/ or phenomenology/ or qualitative analysis/ or grounded theory/ or thematic analysis/ 241053

13 (Qualitative or Interview* or phenomenolog* or Experience* or themes or thematic or ethnograph* or "Focus Group*" or "grounded theor*" or hermeneutic*).mp. 2788931

14 12 or 13 2788931

**15 3 and 4 and 11 and 14 1540**

--

**Notes**

- Default Fields for Unqualified Searches (MP): Searching for a term without specifying a field in Advanced search, or specifying .mp., defaults to the following ‘multi-purpose’ (.mp.) fields for this database: ti,ab,hw,tn,ot,dm,mf,dv,kf,fx,dq.
- KF Keyword Heading Word [Word Indexed]^[[1]](#footnote-1)^
- HW Heading Word [Word Indexed]
- / Exact subject heading – exp / includes narrowing subject headings
- **Search line 2: Corrected from the original search, December 2020, or between schoolchild* teens. Similar also corrected in the other database searches. Corrected also in the first updated search March 2022**
- Or/5-10 = 5 or 6 or 7 or 8 or 9 or 10 or 11

**MEDLINE (Ovid): Ovid MEDLINE(R) ALL <1946 to December 15, 2023>. Date: 18.12.2023**

Link, access is needed: <https://ovidsp.ovid.com/ovidweb.cgi?T=JS&NEWS=N&PAGE=main&SHAREDSEARCHID=1CA5YwjGDIqhyxCBBVG7hcOe4MWUHfbfu3M42SNfVT1lnahU1DpaahPIFbFH8D59Y>

1 adolescent/ or child/ 3171801

2 (adolescen* or boy or boys or child* or girl* or girls* or juvenil* or kid or kids or schoolchild* or teens or teenager* or young* or youth*).ti,ab,kf. 2745418

3 1 or 2 4471747

4 asthma*.ti,ab,hw,kf. 205867

5 exp Exercise/ or Motor Activity/ 341211

6 exp Sports/ 219372

7 Sedentary Behavior/ or (sedentar* or inactiv* or "screen time").ti,ab,kf. 390990

8 (exercise* or "motor activ*" or "physical* activ*" or play or playing or playtime or sport* or training).ti,ab,kf. 1921022

9 or/5-8 2425994

10 Qualitative research/ or Focus Groups/ or Hermeneutics/ or Grounded Theory/ 110539

11 (Qualitative or Interview* or phenomenolog* or Experience* or themes or thematic or ethnograph* or "Focus Group*" or "grounded theor*" or hermeneutic*).mp. 2018027

12 10 or 11 2018027

**13 3 and 4 and 9 and 12 802**

**--**

**Notes**

- KF: Keyword Heading Word [Word Indexed] The Keyword Heading Word (KF) index allows you to retrieve every Keyword Heading assigned by authors that include a particular word. Do this by searching a single word in the Keyword Heading Word (KF) field.
- HW: The Subject Heading Word (HW) index allows you to retrieve every MeSH subject heading and Publication Type that include a particular word. Do this by searching a single word in the Subject Heading Word (HW) field. [Word Indexed]
- MP: Default Fields for Unqualified Searches (MP): Searching for a term without specifying a field in Advanced search, or specifying .mp., defaults to the following ‘multi-purpose’ (.mp.) fields for this database: ti,ab,ot,nm,hw,fx,kf,ox,px,rx,ui,sy.
- MeSH: child, preschool Scope note: 2-5 years, not used
- Search line 2, corrected Se notes from EMBASE search strategy.

**APA PsycInfo (Ovid): APA PsycInfo <1806 to December Week 1 2023>. Date 18.12.2023**

Link, access is needed <https://ovidsp.ovid.com/ovidweb.cgi?T=JS&NEWS=N&PAGE=main&SHAREDSEARCHID=5cXZfkQyo1ZajkjNREGeaCIAKW4HwEBmEa3A1rVxWVlVVMWhQghCnMFLbyqEkRx5f>

1 ("180" or "200").ag. 685256

2 (adolescen* or boy or boys or child* or girl* or girls* or juvenil* or kid or kids or schoolchild* or teens or teenager* or young* or youth*).ti,ab,id. 1216966

3 1 or 2 1414455

4 asthma*.ti,ab,hw,id. 8732

5 exp exercise/ or exp physical activity/ 54807

6 exp Sports/ 44603

7 sedentary behavior/ or screen time/ or (sedentar* or inactiv* or "screen time").ti,ab,id. 27473

8 (exercise* or "motor activ*" or "physical* activ*" or play or playing or playtime or sport* or training).ti,ab,id. 621893

9 or/5-8 651174

10 Metasynthesis.md. 1311

11 Qualitative Study.md. 319896

12 interview.md. 364299

13 exp qualitative methods/ 21582

14 exp qualitative research/ 21582

15 Phenomenology/ or ethnography/ or Hermeneutics/ 31733

16 interview*.ti,ab,hw,id. 400401

17 narrative analysis/ or content analysis/ 7135

18 (Qualitative or phenomenolog* or Experience* or themes or thematic or ethnograph* or "Focus Group*" or "grounded theor*" or hermeneutic*).ti,ab,id. 1016269

19 or/10-18 1357137

**20 3 and 4 and 9 and 19 205**

--

**Note**

- Age group: AG Age Group [Phrase Indexed]
  - 180.ag. school age 6 12 yrs.ag.
  - 200.ag. adolescence 13 17 yrs.ag.
- ID: The Key Concepts (ID) field concisely summarizes a document's subject content. Indexers use the Key Concepts to supplement Subject Headings (SH). Word Indexed
- The Textword (TW) field is an alias for all of the fields in the database that contain text and are appropriate for a free-text subject search. The Text word fields in APA PsycInfo® include Table of Contents (TC), Title (TI), Abstract (AB), and Key Concepts (ID).
- MD: Methodology [Phrase Indexed]. The Methodology (MD) field contains the specific kind of methodology used in a research study. Both the code and the term are searchable. Qualitative Study.md. (1600.md), Metasynthesis.md. (1300.md)
- Exp / - includes narrowing subject headings: S13:
  - Focus Group [+NT]
  - Grounded Theory
  - Interpretative Phenomenological Analysis
  - Narrative Analysis – (also included in S17)
  - Semi-Structured Interview
  - Thematic Analysis
- Search line 2, corrected. See notes EMBASE.

**CINAHL (EBSO*host*). Date 18.12.2023**

Interface - EBSCOhost Research Databases. Search Screen - Advanced Search. Database – CINAHL
Search modes - Boolean/Phrase. Limit is not applied. [Link](https://search.ebscohost.com/login.aspx?direct=true&db=cin20&bquery=(((MH+%26quot%3bQualitative+Studies%26quot%3b)+OR+(MH+%26quot%3bAction+Research%26quot%3b)+OR+(MH+%26quot%3bEthnographic+Research%26quot%3b)+OR+(MH+%26quot%3bEthnological+Research%26quot%3b)+OR+(MH+%26quot%3bEthnonursing+Research%26quot%3b)+OR+(MH+%26quot%3bGrounded+Theory%26quot%3b)+OR+(MH+%26quot%3bNaturalistic+Inquiry%26quot%3b)+OR+(MH+%26quot%3bPhenomenological+Research%26quot%3b)+OR+(MH+%26quot%3bAudiorecording%26quot%3b))+OR+((MH+%26quot%3bThematic+Analysis%26quot%3b)+OR+(MH+%26quot%3bMeta+Synthesis%26quot%3b)+OR+(MH+%26quot%3bContent+Analysis%26quot%3b))+OR+(qualitative+OR+interview*+OR+phenomenolog*+OR+experience*+OR+themes+OR+thematic+OR+narrat*+OR+Ethnograph*+OR+%26quot%3baction+research*%26quot%3b+OR+ehtnonurs*+OR+%26quot%3bgrounded+theor*%26quot%3b+OR+hermeneutic*+OR+%26quot%3bfocus+group*%26quot%3b))+AND+((adolescen*+OR+boy+OR+boys+OR+child*+OR+girl*+OR+girls*+OR+juvenile*+OR+kid+OR+kids+OR+schoolchild*+OR+teens+OR+teenager*+OR+young*+OR+youth*)+AND+asthma*+AND+(((MH+%26quot%3bPhysical+Activity%26quot%3b)+OR+(MH+%26quot%3bSports%2b%26quot%3b))+OR+((MH+%26quot%3bExercise%2b%26quot%3b))+OR+(exercise*+OR+%26quot%3bmotor+activ*%26quot%3b+OR+%26quot%3bphysical+activ*%26quot%3b+OR+play+OR+playing+OR+playtime+OR+sport*+OR+training)+OR+((MH+%26quot%3bLife+Style%2c+Sedentary%26quot%3b)+OR+(MH+%26quot%3bScreen+Time%26quot%3b))+OR+(inactiv*+OR+sedentar*+OR+%26quot%3bscreen+time%26quot%3b)))&type=1&searchMode=Standard&site=ehost-live) (Note access is needed)

**Note:**

- *“The default fields for unqualified searches consist of the following: Title, Abstract and Subject headings”*.^[[2]](#footnote-2)^
- MH = exact subject headings, + includes narrowing headings

| **#** | **Query** | **Results** |
| --- | --- | --- |
| S1 | (MH "Qualitative Studies") OR (MH "Action Research") OR (MH "Ethnographic Research") OR (MH "Ethnological Research") OR (MH "Ethnonursing Research") OR (MH "Grounded Theory") OR (MH "Naturalistic Inquiry") OR (MH "Phenomenological Research") OR (MH "Audiorecording") | 206,375 |
| S2 | (MH "Thematic Analysis") OR (MH "Meta Synthesis") OR (MH "Content Analysis") | 124,261 |
| S3 | qualitative OR interview* OR phenomenolog* OR experience* OR themes OR thematic OR narrat* OR Ethnograph* OR "action research*" OR ehtnonurs* OR "grounded theor*" or hermeneutic* or "focus group*" | 980,373 |
| S4 | S1 OR S2 OR S3 | 997,448 |
| S5^[[3]](#footnote-3)^ | adolescen* or boy or boys or child* or girl* or girls* or juvenile* or kid or kids or schoolchild* or teens or teenager* or young* or youth* | 1,577,536 |
| S6 | asthma* | 50,468 |
| S7 | (MH "Physical Activity") OR (MH "Sports+") | 139,478 |
| S8 | (MH "Exercise+") | 129,677 |
| S9 | exercise* or "motor activ*" or "physical activ*" or play or playing or playtime or sport* or training | 711,768 |
| S10 | (MH "Life Style, Sedentary") OR (MH "Screen Time") | 11,353 |
| S11 | inactiv* OR sedentar* OR "screen time" | 43,922 |
| S12 | S7 OR S8 OR S9 OR S10 OR S11 | 769,542 |
| S13 | S5 AND S6 AND S12 | 2,261 |
| S14 | S4 AND S13 | **361** |

**SPORTDiscus (EBSCO*host*). Date 18.12.2023**

Interface - EBSCOhost Research Databases. Search Screen - Advanced Search. Database - SPORTDiscus

Search modes - Boolean/Phrase. Limit is not applied. [Link](https://search.ebscohost.com/login.aspx?direct=true&db=sph&bquery=(adolescen*+OR+boy+OR+boys+OR+child*+OR+girl*+OR+girls*+OR+juvenile*+OR+kid+OR+kids+OR+schoolchild*+OR+teens+OR+teenager*+OR+young*+OR+youth*)+AND+asthma*+AND+(Qualitative+OR+Interview*+OR+phenomenolog*+OR+Experience*+OR+themes+OR+thematic+OR+ethnograph*+OR+%26quot%3bFocus+Group*%26quot%3b+OR+%26quot%3bgrounded+theor*%26quot%3b+OR+hermeneutic*)&type=1&searchMode=Standard&site=ehost-live) (Access is needed)

Note: “*The default fields for unqualified searches consist of the following: Title, Author, Corporate Author, Conference, Source, Publisher, Abstract, Keywords, Subject Classification, and Handicapped Codes”^[[4]](#footnote-4)^*

| **#** | **Query** | **Results** |
| --- | --- | --- |
| S1 | adolescen* or boy or boys or child* or girl* or girls* or juvenile* or kid or kids or schoolchild* or teens or teenager* or young* or youth* | 285,672 |
| S2 | asthma* | 4,572 |
| S3 | Qualitative or Interview* or phenomenolog* or Experience* or themes or thematic or ethnograph* or "Focus Group*" or "grounded theor*" or hermeneutic* | 201,539 |
| S4 | S1 AND S2 AND S3 | **139** |

**SocINDEX (EBSCO*host*). Date 18.12.2023**

Interface - EBSCOhost Research Databases. Search Screen - Advanced Search. Database - SocINDEX. Search modes - Boolean/Phrase. Limit is not applied. [Link](https://search.ebscohost.com/login.aspx?direct=true&db=snh&bquery=(adolescen*+OR+boy+OR+boys+OR+child*+OR+girl*+OR+girls*+OR+juvenile*+OR+kid+OR+kids+OR+schoolchild*+OR+teens+OR+teenager*+OR+young*+OR+youth*)+AND+asthma*+AND+(exercise*+OR+%26quot%3bmotor+activ*%26quot%3b+OR+%26quot%3bphysical*+activ*%26quot%3b+OR+play+OR+playing+OR+playtime+OR+sport*+OR+training+OR+sedentar*+OR+inactiv*+OR+%26quot%3bscreen+time%26quot%3b)+AND+(Qualitative+OR+Interview*+OR+phenomenolog*+OR+Experience*+OR+themes+OR+thematic+OR+ethnograph*+OR+%26quot%3bFocus+Group*%26quot%3b+OR+%26quot%3bgrounded+theor*%26quot%3b+OR+hermeneutic*)&type=1&searchMode=Standard&site=ehost-live) (Access is needed)

NOTE: “*The default fields for unqualified searches consist of the following: all authors, all subjects, all keywords, all title info (including source title) and all abstracts”*.^[[5]](#footnote-5)^

| **#** | **Query** | **Results** |
| --- | --- | --- |
| S1^[[6]](#footnote-6)^ | adolescen* or boy or boys or child* or girl* or girls* or juvenile* or kid or kids or schoolchild* or teens or teenager* or young* or youth* | 543,929 |
| S2 | asthma* | 2,568 |
| S3 | exercise* or "motor activ*" or "physical* activ*" or play or playing or playtime or sport* or training OR sedentar* OR inactiv* OR "screen time" | 214,034 |
| S4 | Qualitative or Interview* or phenomenolog* or Experience* or themes or thematic or ethnograph* or "Focus Group*" or "grounded theor*" or hermeneutic* | 470,797 |
| S5 | S1 AND S2 AND S3 AND S4 | **25** |

**Social Sciences Citation Index (Web of Science (WoS)). Date 18.12.2023**

# Searches^[[7]](#footnote-7)^:

1: TS=(adolescen* or boy or boys or child* or girl* or girls* or juvenil* or kid or kids or schoolchild* or teens or teenager* or young* or youth*) AND TS=asthma* AND TS=(exercise* or "motor activ*" or "physical activ*" or play or playing or playtime or sport* or training or sedentar* OR inactiv* OR "screen time") AND TS=(Qualitative or Interview* or phenomenolog* or Experience* or themes or thematic or ethnograph* or "Focus Group*" or "grounded theor*" or hermeneutic*)

**Editions: WOS.SSCI**

Date Run: Mon Dec 18 2023

Results: **307**

Link (Access is needed)

<https://www.webofscience.com/wos/woscc/summary/97ddc8f5-b467-42d6-bca7-0fd8b06c7fd9-2ba1e322/relevance/1>

**ProQuest: Healthcare Administration Database, Nursing & Allied Health Database, Public Health Database**^[[8]](#footnote-8)^ **Date: 18.12.2023**

noft(adolescen* or boy or boys or child* or girl* or girls* or juvenil* or kid or kids or schoolchild* or teens or teenager* or young* or youth*) AND noft(asthma*) AND noft(exercise* or "motor activ*" or "physical activ*" or play or playing or playtime or sport* or training or sedentar* OR inactiv* OR "screen time")

Limit: Dissertation & Thesis: **58 hits** (18.12.2023)

Link, Access is needed <https://www.proquest.com/search/2120278?accountid=45259>

**Notes**

APA PsycInfo & CINAHL also includes dissertations

--

**Result:**

Total record retrieved 18.12.2023: 3437

Total duplicates removed through EndNote 21^[[9]](#footnote-9)^:1251

Total unique records:2186

1. Previous field code name for this field was: KW used in the original search December 2020. The KW filed is now Phrase index, previously word indexed like the KF field [↑](#footnote-ref-1)
2. <http://support.ebsco.com/help/?int=ehost&lang=en&feature_id=Databases&TOC_ID=Always&SI=0&BU=0&GU=1&PS=0&ver=live&dbs=cin20jnh,cin20> [↑](#footnote-ref-2)
3. Corrected from the original search (December 2020), OR between schoolchild* teens* The first updated search march 2022 this were corrected, so no difference between the search March 2022 and December 2023. [↑](#footnote-ref-3)
4. <http://support.ebsco.com/help/?int=ehost&lang=en&feature_id=Databases&TOC_ID=Always&SI=0&BU=0&GU=1&PS=0&ver=live&dbs=,sph> [↑](#footnote-ref-4)
5. <http://support.ebsco.com/help/?int=ehost&lang=en&feature_id=Databases&TOC_ID=Always&SI=0&BU=0&GU=1&PS=0&ver=live&dbs=,snh> [↑](#footnote-ref-5)
6. Corrected from the original search, OR between schoolchild* teens* [↑](#footnote-ref-6)
7. Corrected from the original search, OR between schoolchild* teens*, as noted for EMBASE. [↑](#footnote-ref-7)
8. Corrected from the original search, OR between schoolchild* teens*, as noted for EMBASE. [↑](#footnote-ref-8)
9. Duplicate records were removed through EndNote 21, in a stepwise process inspired by Bramer et al., unpublished version by the University Library at Karolinska Institutet <https://kib.ki.se/en/node/1379> [↑](#footnote-ref-9)
