# Supplementary material for: Physical Activity Enforces Well-being or Shame in Children and Adolescents With Asthma: A Meta-ethnography
Source: Inquiry. 2024 Nov 5;61:00469580241290086. doi: 10.1177/00469580241290086 (PMC11536505; doi:10.1177/00469580241290086)
Supplement: sj-docx-2-inq-10.1177_00469580241290086 – Supplemental material for Physical Activity Enforces Well-being or Shame in Children and Adolescents With Asthma: A Meta-ethnography [file sj-docx-2-inq-10.1177_00469580241290086.docx]

**Backward and forward citations. Reference list and cited by for the included studies. Date: 2022-03-23. Update cited by check, 2023-12-19.**

Sources:

- Scopus database is used for references indexed in this database, both reference list and cited by.
- GS is used for dissertation/Master thesis, manually checking cited by references, and manually screened. Dissertation/Master thesis reference list is checked manually, in the process of the full-text reading of the document.

|  | Study | Scopus **Reference list:**  2022-03-23 | Scoups **Cited by**  2022-03-23 | GS  Cited by.  2022-03-23 | Scopus, Cited by. 2023-12-19 | GS Cited by. 2023-12-19 |
| --- | --- | --- | --- | --- | --- | --- |
| 1 | Brynjulfsen T, Demmelmaier I, Berntsen S, Foyn TH, Andersen OK, Stang J, et al. Motivation for physical activity in adolescents with asthma. Journal of Asthma. 2020:1-9. Updated article information: 2021, vol: 58, Issue 9. Doi:10.1080/02770903.2020.1778025 | 39 | 0 |  | 5 |  |
| 2 | Cardwell FS, Elliott SJ. They say 'kids with asthma don't play sports': geographies of asthma. Childrens Geographies. 2019;17(6):649-63. DOI:10.1080/14733285.2019.1649362 | 31 | 0 |  | 0 |  |
| 3 | Fereday J, MacDougall C, Spizzo M, Darbyshire P, Schiller W. 'There's nothing I can't do--I just put my mind to anything and I can do it': a qualitative analysis of how children with chronic disease and their parents account for and manage physical activity. BMC Pediatrics. 2009;9. DOI:10.1186/1471-2431-9-1 | 52 | 41 |  | 44 |  |
| 4 | Jago R, Searle A, Henderson AJ, Turner KM. Designing a physical activity intervention for children with asthma: A qualitative study of the views of healthcare professionals, parents and children with asthma. BMJ Open. 2017;7(3):e014020. DOI:10.1136/bmjopen-2016-014020 | 31 | 11 |  | 15 |  |
| 5 | Lack S, Schechter MS, Everhart RS, Thacker Ii LR, Swift-Scanlan T, Kinser PA. A mindful yoga intervention for children with severe asthma: A pilot study. Complementary therapies in clinical practice. 2020;40:101212. DOI: 10.1016/j.ctcp.2020.101212 | 58 | 0 |  | 1 |  |
| 6 | Protudjer JLP. Talking to children about lifestyle, weight and asthma: A substudy of the Gender-Related Evolution of Asthma Team:interdisciplinary capacity enhancement (GREATice) [Master of Science]. Ann Arbor: University of Manitoba (Canada); 2007. |  |  | 0 |  | 0 |
| 7 | . Protudjer JLP. Weight, Related Lifestyle Behaviours and Asthma in Manitoba Children. Ann Arbor: University of Manitoba (Canada); 2012. |  |  | 0 |  | 0 |
| 8 | Protudjer JLP, McGavock JM, Ramsey CD, Sevenhuysen GP, Kozyrskyj AL, Becker AB. "Asthma isn't an excuse, it's just a condition": Youths' perceptions of physical activity and screen time. Journal of Asthma. 2012;49(5):496-501. DOI: 10.3109/02770903.2012.680637 | 29 | 8 |  | 8 |  |
| 9 | Shaw MR. Perceptions of exercise among school aged children with asthma, Thesis, University of Arizona; 2010. [see also article below] |  |  | 0 |  | 0 |
| 10 | Shaw MR, Davis AHT. Perceptions of exercise among school-aged children with asthma. Journal of Asthma and Allergy Educators. 2011;2(5):233-40. DOI: 10.1177/2150129710397885 | 25 | 5 |  | 4 |  |
| 11 | Shaw MR, Katz J, Benavides-Vaello S, Oneal G, Holliday C. Views on exercise: A grounded theory exploration of the creation of exercise perceptions in Hispanic children with asthma. Hispanic Health Care International. 2017;15(2):71-8. DOI:10.1177/1540415317707915 | 36 | 3 |  | 4 |  |
| 12 | Spencer-Cavaliere N, Watkinson EJ. Inclusion understood from the perspectives of children with disability. Adapted Physical Activity Quarterly. 2010;27(4):275-93. DOI: 10.1123/apaq.27.4.275 | 46 | 112 |  | 145 |  |
| 13 | Walker TJ, Reznik M. In-school asthma management and physical activity: Children's perspectives. Journal of Asthma. 2014;51(8):808-13. DOI: 10.3109/02770903.2014.920875 | 34 | 32 |  | 38 |  |
| 14 | Westergren T, Fegran L, Nilsen T, Haraldstad K, Kittang OB, Berntsen S. Active play exercise intervention in children with asthma: A PILOT STUDY. BMJ Open. 2016;6(1):e009721. DOI: 10.1136/bmjopen-2015-009721 | 45 | 14 |  | 21 |  |
| 15 | Williams B, Hoskins G, Pow J, Neville R, Mukhopadhyay S, Coyle J, et al. Low exercise among children with asthma: a culture of over protection? A qualitative study of experiences and beliefs. British Journal of General Practice. 2010;60(577):319-26. DOI: 10.3399/bjgp10X515070 | 39 | 32 |  | 41 |  |
| 16 | Winn CON, Mackintosh KA, Eddolls WTB, Stratton G, Wilson AM, Rance JY, et al. Perceptions of asthma and exercise in adolescents with and without asthma. Journal of Asthma. 2018;55(8):868-76. DOI: 10.1080/02770903.2017.1369992 | 47 | 21 |  | 32 |  |
| 17 | Jordan KA, Mackintosh KA, Davies GA, Griffiths CJ, Lewis PD, McNarry MA: **Perceptions surrounding the possible interaction between physical activity, pollution and asthma in children and adolescents with and without asthma**. *BMC Public Health* 2023, **23**(1):2416 | 42 |  |  | 0 (22.02.24) | 0 (22.02.24) |
| 18 | Koskela-Staples NC, Yourell JL, Fedele DA, Doty J: **Physical Activity Engagement: Perspectives From Adolescents With Comorbid Asthma and Overweight/Obesity and Their Caregivers**. *J Pediatr Psychol* 2023, **48**(8):707-719 | 55 |  |  | 0 (22.02.24) | 0 (22.02.24) |

**Result 2022-03-23:**

- Total: 823, both records from reference list and record cited the included studies.
- Internal duplicates, checked through EndNote X9.3.3: 141.
- Unique in this group: 682.

**Result 2023-12-19:**

No new included studies from the updated search 2022-03-23, thus only update the cited by records.

- Total cited by records: 358.
- Internal duplicates, removed through EndNote 21:38
- Unique in this group: 320.

**Result reference list 2024-02-22**

Two included studies from updated search

- Total references: 93.
