# Supplementary material for: Physical Activity Enforces Well-being or Shame in Children and Adolescents With Asthma: A Meta-ethnography
Source: Inquiry. 2024 Nov 5;61:00469580241290086. doi: 10.1177/00469580241290086 (PMC11536505; doi:10.1177/00469580241290086)
Supplement: sj-docx-3-inq-10.1177_00469580241290086 – Supplemental material for Physical Activity Enforces Well-being or Shame in Children and Adolescents With Asthma: A Meta-ethnography [file sj-docx-3-inq-10.1177_00469580241290086.docx]

**Supplementary file 3; Table 1**. Study characteristics of the included studies.

| **Author, year,**  **Area, country** | **Participants with asthma; n, age, characteristics** | **Setting** | **Phenomenon of Interest** | **Methodology,**  **Method** | **Data analysis** |
| --- | --- | --- | --- | --- | --- |
| **Brynjulfsen et al.**, 2020,  Norway | 18, 13−17 yrs, 33% overweight, 71% Caucasian | Aten-week play-based exercise intervention | Motivation for PA and exercise | Qualitative,  Focus group interviews & field observations | Thematic deductive analysis (Braun & Clarke, 2006) |
| **Cardwell & Elliott**, 2019,  Ontario, Canada | 11, 11−18 yrs, team sports participants, diverse SES | Youth team sports | The knowledge, attitudes, and practices (e.g., management behaviors) of organized youth team sport athletes | Qualitative from a geographical/environmental perspective,  Semi-structured in-depth interviews | Transcripts were coded deductively, and inductively (Nowell et al., 2017) |
| **Fereday et al.**, 2009,  South Australia | 14, 4−15 yrs, NR | Chronic disease & tertiary pediatric hospitals | Experiences and perceptions of physical activity, exercise, sport and play and physical activity's barriers and enablers. | Interpretive phenomenology  Focus groups, mapping & photovoice | Codes were then clustered into themes and sorted |
| **Jago et al.**, 2017,  England, UK | 9, 6–7 yrs, NR | Primary care practices | Reasons why children with asthma are less active and how a physical activity program could be developed | Qualitative  In-depth interviews | Independently coding, discuss coding, agree coding frame (Braun & Clarke, 2006). |
| **Jordan et al.**, 2023,  Wales, UK | 11, 7−17 yrs, NR | Diverse geographical locations during Covid -19 | Perceptions regarding asthma, air pollution, PA and their interaction | Qualitative,  Semi structured individual interviews | Inductive and deductive loosely connected to Braun & Clarke´s thematic analysis (2006) |
| **Koskela-Staples et al.**, 2023,  Florida, US | 20, 13−18 yrs, overweight/obese, 9 Black, 7 White, 2 Hispanic, 2 Multiracial | Local clinics and local community. Comorbidity asthma - overweight/obesity | Influences, processes, and behaviors across domains that relate to PA engagement | Qualitative,  Separated semi structured interviews | Braun & Clarke´s thematic analysis (2012) |
| **Lack et al.**, 2020,  East coast, US | 15, 7–11 yrs, NR | A mindful yoga information / urban medical center | Acceptability of the mindful yoga intervention | Mixed methods approach with an embedded design / qualitative descriptive,  Semi-structured interview | Coding / interpretation of quantitative counts of codes, providing themes (Sandelowski, 2000) |
| **Protudjer**, 2007,  Manitoba, Canada | 22, 11–12 yrs, 4/5 Caucasian | The SAGE/GREATice cohort and case-control study / participants homes or the allergy lab | Perceptions of weight and dieting, healthful eating and physical activity & living and coping with disease. | Qualitative,  In-depth semi structured interviews | Organizing, generating categories / nodes, themes and patterns; looking for overarching themes whilst considering alternative explanations (Patton, 2002). |
| **Protudjer**, 2012 / **Protudjer et al.**, 2012,  Manitoba, Canada | 22, 15−16 yrs, NR | Manitoba Birth Cohort study and the Canadian Asthma Primary Prevention Study | Lived experience and perceptions of physical activity and screen time by asthma status | Qualitative,  Focus group and individual interviews | Constant comparative method, rooted in a pragmatic worldview (Creswell, 2007). |
| **Shaw**, 2010 / **Shaw & Davis** 2011,  Arizona, US | 10, 8−12 yrs, 8 Caucasian, 1 half Chinese, 1 African American | Intermittent to severe-persistent asthma, family income between $20-60.000, minimum some college education, home environment | Explicate predominant concepts in perception of exercise and relationships among predominant concepts | Grounded theory  Theoretical sampling / individual semi structured interviews | Grounded theory (Corbin & Strauss, 2008); Iterative data analysis; coding, comparative analysis, open coding, theoretical integration, and theory refinement. |
| **Shaw et al.**, 2017,  Washington state, US | 8, 8−14 yrs, Hispanic | Participants’ homes | A previously developed grounded theory called The Process of Creating Perceptions of Exercise | Grounded theory,  Theoretical sampling / Individual interviews | Grounded theory iterative data analysis including: (a) beginning coding, (b) open coding, (c) comparative analysis, (d) theoretical integration, and (e) theory refinement (Corbin & Strauss, 2015) |
| **Spencer-Cavaliere & Watkinson**, 2010,  Alberta/Manitoba, Canada | 1, 9 yrs, NR | Chronic conditions / Specialized sport and activity programs | Inclusion in physical activity as a subjective experience | Idiographic explorative and descriptive,  Individual semi structured interviews and reflective fieldnotes | Content analysis identifying core consistencies and meanings (Patton, 2002). |

Abbreviations: yrs; years, SES; socioeconomic status, NR; not reported

**Supplementary file 3; Table 1.** (continued).

| **Author, year,**  **Area, country** | **Participants with asthma; n, age, characteristics** | **Setting** | **Phenomenon of Interest** | **Methodology,**  **Method** | **Data analysis** |
| --- | --- | --- | --- | --- | --- |
| **Walker & Reznik**, 2014,  New York, US | 23, 8−10 yrs, 56% overweight/obese, 13 not well controlled/very poorly controlled, Minority | Inner city elementary schools | Perceptions of how in-school asthma management impacts PA | Qualitative,  Individual interviews | Thematic and content analysis (Joffe & Yardley, 2003) |
| **Westergren et al.**, 2016,  Norway | 6, 10–12 yrs, 1 overweight | A 6-week exercise intervention designed as active play for children with asthma | Perceptions of participating in a 6-week exercise intervention designed as active play for children with asthma. | Convergent parallel mixed-methods design,  Semi structured focus groups and field observations | Systematic text condensation (Malterud, 2012) |
| **Williams et al.**, 2010,  Scotland, UK | 30, 6−14 yrs, NR | General practitioners’ practices | Ways in which beliefs about asthma and exercise influenced the child’s willingness to engage in physical activity | Qualitative,  Individual interviews including drawings | Constant comparative technique (Patton, 2001) |
| **Winn et al.**, 2018,  UK | 26, 11−14 yrs, NR | Secondary schools as part of a wider randomized control trial (the X4A trial: eXercise for Asthma with Commando Joe’s) | Views about exercise and asthma, and the perceived benefits of and barriers to participation. | Qualitative,  Formative/semi structured group interviews | Thematically analyzed in a deductive manner and presented via diagrams of key emergent themes (pen profiles) |

Abbreviations: yrs; years, SES; socioeconomic status, NR; not reported

**Supplementary file 3; Table 2**. Critical appraisal of the included studies included share of ‘yes’ in each study an in each item across studies.

| Author, year | Q1 | Q2 | Q3 | Q4 | Q5 | Q6 | Q7 | Q8 | Q9 | Q10 | *%* |
| --- | --- | --- | --- | --- | --- | --- | --- | --- | --- | --- | --- |
| Brynjulfsen et al., 2020 | Y | Y | Y | Y | Y | Y | Y | Y | Y | Y | *100* |
| Cardwell & Elliot, 2019 | Y | Y | Y | Y | Y | Y | N | Y | Y | Y | *90* |
| Fereday et al., 2009 | Y | Y | Y | Y | Y | Y | Y | Y | Y | Y | *100* |
| Jago et al., 2017 | Y | Y | Y | Y | Y | Y | N | Y | Y | Y | *90* |
| Jordan et al., 2023 | Y | Y | Y | Y | Y | N | N | Y | Y | Y | *80* |
| Koskela-Staples et al., 2023 | Y | Y | Y | Y | Y | Y | Y | Y | Y | Y | *100* |
| Lack et al., 2020 | Y | Y | Y | Y | Y | N | N | Y | Y | Y | *80* |
| Protudjer, 2007 | Y | Y | Y | Y | Y | Y | Y | Y | Y | Y | *100* |
| Protudjer, 2012 | Y | Y | Y | Y | Y | Y | Y | Y | Y | Y | *100* |
| Protudjer et al., 2012 | Y | Y | Y | Y | Y | Y | N | Y | Y | Y | *90* |
| Shaw, 2010 | Y | Y | Y | Y | Y | Y | Y | Y | Y | Y | *100* |
| Shaw & Davis, 2011 | Y | Y | Y | Y | Y | Y | N | Y | N | Y | *80* |
| Shaw et al., 2017 | Y | Y | Y | Y | Y | Y | N | Y | Y | Y | *90* |
| Spencer-Cavaliere & Watkinson, 2010 | Y | Y | Y | Y | Y | Y | Y | Y | Y | Y | *100* |
| Walker & Reznik, 2014 | Y | Y | Y | Y | Y | N | N | Y | Y | Y | *80* |
| Westergren et al., 2016 | Y | Y | Y | Y | Y | Y | Y | Y | Y | Y | *100* |
| Williams et al., 2010 | Y | Y | Y | Y | Y | Y | N | Y | Y | Y | *90* |
| Winn et al., 2018 | Y | Y | Y | Y | Y | N | N | Y | Y | Y | *80* |
| *Share of studies with a ‘yes’ (%)* | *100* | *100* | *100* | *100* | *100* | *78* | *44* | *100* | *94* | *100* |  |

Q1. Congruity between the stated philosophical perspective and the research methodology, Q2. Congruity between the research methodology and the research question or objectives, Q3. Congruity between the research methodology and the methods used to collect data, Q4. Congruity between the research methodology and the representation and analysis of data, Q5. There is congruence between the research methodology and the interpretation of results, Q6. Locating the researcher culturally or theoretically, Q7. Influence of the researcher on the research, and vice-versa, is addressed, Q8. Representation of participants and their voices, Q9. Ethical approval by an appropriate body, Q10. Relationship of conclusions to analysis, or interpretation of the data

**Supplementary file 3; Table 3.** Grid of 3^rd^ order interpretations / reciprocal translations (column headings) describing children’s and adolescents’ experiences with participation in, or limitations of, PA from coding of 1^st^ and 2^nd^ order interpretations (metaphors, column rows) in studies included.

| **Author, year, dominating perspective*** | **Feeling related to and connected with friends and family in PA** | **Acquiring and managing new PA and asthma skills** | **Enjoying PA and experiencing well-being** | **Feeling misunderstood and penalized in relation to PA** | **Experiencing nervousness, embarrassment, shame, and sadness during PA** | **Withdrawing from PA due to asthma, environment, and/or socially imposed attitudes** |
| --- | --- | --- | --- | --- | --- | --- |
| **Cardwell & Elliott** 2019  (Index study), Balanced | Awareness and recognition are supporting / Value understanding, support, and help to calm down | (Emergency) medications and water brakes to manage | Sports and play make stress go away | Disappointing teammates and coach / Stigmatization and being penalized by teammates and coaches | Embarrassment when using medication or reducing participation due to symptoms | Exercise triggers asthma / Go off and feeling panicked by symptoms / Come off the field due to temperature, humidity, season, and allergy |
| **Fereday et al**. 2009, Asthma does not restrain PA | Having special friends for support / No children were teased | Asthma do not hinder PA / Stopping, resting, and medicate to overcome disruptions of PA / Participating in wide variety of PA and sports | Loving sports | Being screamed at when just can’t keep going / Being singled out and treated differently / Teachers do not understand |  | Cold weather and allergy trigger asthma / Hospitalizations deter PA |
| **Jago et al**. 2017, Asthma does restrain PA |  | Suggesting increasing inhaler use facilitate PA |  |  | Worry about feeling different / Reluctance to ask for inhaler | Asthma stops participation in sports / Just sit down instead of asking for inhaler / Forgetting (brown) puffer limits PA / Limited access to inhaler at school |
| **Williams et al**. 2010 ], Asthma does restrain PA | Sports promote a sense of community, belonging and acceptance | Beliefs about capability, safety, and motivation influence PA / Using inhaler and rest, to then keep it going | Games and sports are fun | Strong negative sanctions related to limited abilities of full participation | Fear of friends laugh in onset of symptoms | Onset of breathlessness as a limit of capability / Safety concerns about exercise as a threat also exposing them to environmental triggers / Breathlessness of exertion ascribed to asthma / Believing breathlessness is a signal of ‘overexertion’ |
| **Lack et al**. 2020, Asthma does not restrain PA | Might be willing of yoga with friends | Yoga does not make it hard to breath | Yoga is good, fun, great and relaxing / Feeling calm, happy, confident, and mindful |  | Becoming self-conscious and embarrassed during yoga | Asthma stops playing sports / Girls more likely to scale down PA to prevent symptoms |
| **Jordan et al**. 2023, Asthma does restrain PA |  | PA is an asthma management strategy / Take blue inhaler before PA |  |  |  | Consider severity when encouraging PA / PA in polluted area cause more harm than good |
| **Shaw** 2010, **Shaw & Davis**, 2011, Balanced | Exercise with friends is social benefits and sharing interests | Striving for normalcy to fit in by keeping asthma private, planning, and continuing / Adjusting individually to tolerated intensity and duration / Learning to tolerate / Continuing requires planning / Planning exercise for normalcy / Parents, PE teachers, and HCPs influence exercise perceptions / Creation of perceptions of exercise is ongoing process / Parents support and are (the most important among) role models / PE teachers teach importance of exercise / Remaining active despite triggers, symptoms, and tolerated activity level | Exercise and PA is fun and improves health |  | Keeping asthma private to PE teachers, coaches, and friends in fear of being thought of as different or unable | Symptoms and side aches all of the time making me feel I can’t move anymore / Asthma triggers influence exercise and exercise triggers symptoms / Avoiding activities in individual beliefs of not tolerating / Heavy and tired legs / Sore and tired / Wiped out and dizzy airhead / Tight chest / Cough / Wheeze / Throat hurts / Hurt superbad / Feelings / Restrict PA to avoid being different and ‘slow’ |

*Dominating perspective according to reviewers’ interpretation on whether study focus on how asthma restrain PA or not restrain PA.

Abbreviations: PA; physical activity, PE; physical education

**Supplementary file 3; Table 3.** (continued)

| **Authors, year, dominating perspective*** | **Feeling related to and connected with friends and family in PA** | **Acquiring and managing new PA and asthma skills** | **Enjoying PA and experiencing well-being** | **Feeling misunderstood and penalized in relation to PA** | **Experiencing nervousness, embarrassment, shame, and sadness during PA** | **Withdrawing from PA due to asthma, environment, and/or socially imposed attitudes** |
| --- | --- | --- | --- | --- | --- | --- |
| **Shaw et al**. 2017, Asthma do not restrain PA | Sharing experiences with peers with asthma / peers supporting and advocating well-being |  |  |  | Being afraid, embarrassed, and ashamed |  |
| **Walker & Reznik** 2014, Asthma does restrain PA |  | Immediate access to medication makes returning to activity possible |  |  | Nervousness and embarrassment concerning use of medication regardless of gender and weight category | Lack of accessible medication due to school restrictions / Stopping PE because of asthma symptoms / Preventing asthma by sitting out from activity / Stopping PA to control symptoms / Not returning to PE after taking medication at nurses’ office |
| **Spencer-Cavaliere & Watkinson** 2010, Asthma does not restrain PA | Having friends make you feel included in sports and games / Contributing is feeling included / Coach can make me feel included |  |  |  |  |  |
| **Westergren et al**. 2016, Balanced | Exercise together is bonding / Humor and encouragement increases effort and make situation harmless | Participating in different sports / Exhaustion is not due to asthma | Exercise is improved health and fitness / Exercise is easier when fun |  | Do not want attention on / care for asthma | Asthma restricts PA / Always have to stop to breathe / Asthma was the reason for not attending PE classes and causes pain in chest and heart / Loosing lead in competition decreases enjoyment |
| **Protudjer** 2007, Balanced | Favorite PA is source of camaraderie | Choosing anaerobic low-intensity sports / Managing asthma by puffers / Normality is when asthma do not interfere with PA / Participating through adapting / Stressing normality and down-play impact of asthma / You can still do everything despite asthma / Involved in PA to protect identity, be typical, and fit in | Health is eating right and lots of exercise / Favorite PA is source of pleasure / Still lots of fun without going really, really fast / Stay fit to manage asthma |  | Feeling different and weird because of asthma (especially girls) / Feeling embarrassed, or people laughing | Slow down or sit out due to pain, burn, and cough upon exertion |
| **Winn et al**. 2018, Balanced |  | Altering breathing patterns to control asthma / Controlling symptoms by inhaler / Being fitter reduced asthma symptoms / high interval intensity is difficult due to asthma / Increase exercise to show they are not affected | Exercise is a favorite thing / Sports is a common activity and games the most enjoyable / Exercise improves health | Misinterpreted for using asthma as an excuse / Being left out by friends / Struggling laughing with friends |  | Preventing from doing activity because of fear of asthma attack / Reducing involvement and participation in PE lesson / Needing breakes to catch breath / Not taking inhaler prior to activity / Participating less in exercise / Long distance running difficult / Using asthma as an excuse |

*Dominating perspective according to reviewers’ interpretation on whether study focus on how asthma restrain PA or not restrain PA.

Abbreviations: PA; physical activity, PE; physical education

**Supplementary file 3; Table 3.** (continued)

| **Authors, year, dominating perspective*** | **Feeling related to and connected with friends and family in PA** | **Acquiring and managing new PA and asthma skills** | **Enjoying PA and experiencing well-being** | **Feeling misunderstood and penalized in relation to PA** | **Experiencing nervousness, embarrassment, shame, and sadness during PA** | **Withdrawing from PA due to asthma, environment, and/or socially imposed attitudes** |
| --- | --- | --- | --- | --- | --- | --- |
| **Brynjulfsen et al**. 2020, Balanced | Understanding of asthma and relatedness / Sharing and feeling similar / Social support increase effort and motivation |  | Mastering PA is fun, and fun is mastering / Winning is fantastic, competition may motivate | Teachers lack asthma knowledge | Embarrassment of unwanted attention around asthma symptoms and medications | Cannot but wish to keep pace with peers / Pollen and cold air restricted PA |
| **Protudjer** 2012, **Protudjer et al**. 2012, Asthma does not restrain PA |  | Asthma is not an excuse for inactivity / Changing to less aerobic demanding PA and use preventive medication / Controlling asthma to be socially accepted / Acknowledging the role of reliever medication / PA used to be more difficult before | Sport is a matter of interest and remain competent / Sport is possibility for improving / Sport is really important and help you keep focused and reduce stress |  |  | Screen time is important to youths’ communication |
| **Koskela-Staples et al**. 2023, Balanced | Dad and sister helps me get through running / Easier to stay in pace with people at your level / Family support facilitates PA / Having encouraging people promote PA / Motivated by friend going to gym a lot / Mum makes me walk the dog / Mum willing to take me to practice / Playing and do challenges with friends doing push-ups, pull-ups, and everything / Talk about issues with mother and grandmother makes PA easier / Non-sports-activities facilitate PA / Allowed independence facilitates PA & too prompting PA may create opposition / Incentives (from parents) help | Asthma medication enable baseball and things / Clinician-adolescent communication promote PA / Doctors knowing you should provide you awareness and education about PA / Facilities, equipment, and good weather promote PA / Good weather important go engage in PA | Active to lose weight and feel less stressed / Do PA for mental, emotional and physical health / Get in shape to become healthy / Something I want to do, I do / Motivated by tracking diet and workouts on app |  | Anxious and embarrassed doing PA in front of others / Embarrassing having inhaler out / Mental health is biggest barrier / Unmotivated & sad by own weight / Depression gets in the way of PA / Motivation loss / Lazy and do not like to move | Asthma enforce brakes when running / Cramp in stomach slows me down / Heat, cold air, rain, pollen, dust, and air pollution hinder PA / Lack of time for PA / Not able to do physical things because family member broke her neck / Online schooling & Covid-19 stops walking / Poor sleep hinder PA / Stuff going on (in neighborhood) make me not go out by night / Systemic steroids lead to wight gain making PA difficult |

*Dominating perspective according to reviewers’ interpretation on whether study focus on how asthma restrain PA or not restrain PA.

Abbreviations: PA; physical activity, PE; physical education
